# Nanoformulation of a Trypanocidal Drug Isometamidium Chloride Ameliorates the Apurinic-Apyrimidinic DNA Sites/Genotoxic Effects in Horse Blood Cells

**DOI:** 10.3390/jox13010012

**Published:** 2023-03-02

**Authors:** Sandeep Singh, Balvinder Kumar, Neeraj Dilbaghi, Nisha Devi, Minakshi Prasad, Anju Manuja

**Affiliations:** 1 ICAR-National Research Centre on Equines, Hisar 125001, Haryana, India; 2 Department of Bio & Nano Technology, Guru Jambheshwar University of Science & Technology, Hisar 125001, Haryana, India

**Keywords:** isometamidium chloride, *Trypanosoma*, genotoxicity, AP sites, DNA damage, nanoformulation

## Abstract

Isometamidium chloride (ISM) is a trypanocide for the prophylactic and therapeutic use against vector-borne animal trypanosomosis (mainly Surra caused by *Trypanosoma evansi*) and African animal trypanosomosis caused by *T. congolense*/*T. vivax*/*T. brucei*). ISM was found to be an efficient trypanocide for therapeutic/prophylactic use against trypanosomosis; however, it produces some local and systemic detrimental effects in animals. We synthesized isometamidium chloride-loaded alginate gum acacia nanoformulation (ISM SANPS) to lessen the detrimental side effects of isometamidium chloride (ISM) while treating trypanosomal diseases. We intended to determine the cytocompatibility/toxicity, and DNA deterioration/chromosomal structural or number changes (genotoxicity) of ISM SANPs using mammalian cells in a concentration-dependent manner. Apurinic/apyrimidinic (AP) sites are one of the major types of DNA lesions formed during base excision and repair of oxidized, deaminated, or alkylated bases. The intensity of the cellular AP site is an excellent marker of the deterioration of DNA quality. We thought it pertinent to quantify the AP sites in ISM SANPs-treated cells. Our investigations established a dose-dependent cyto-compatibility or toxicity and DNA impairment (genotoxicity) in ISM SANPs-treated horse peripheral blood mononuclear cells. ISM SANPs were biocompatible at various concentrations tested on the mammalian cells.

## 1. Introduction

Isometamidium chloride is a trypanocide for the prophylactic and therapeutic use against animal trypanosomosis (mainly Surra caused by *Trypanosoma evansi* and African animal trypanosomosis caused by *T. congolense*/*T. vivax*/*T. brucei*), mainly transmitted by hematophagous flies. Animal trypanosomosis are diseases of economic importance affecting livestock worldwide [1,2]. Isometamidium chloride (ISM), an old phenanthridium drug, was obtained by the reaction between m-amidino-benzene diazonium chloride and homidium chloride in 1958 [3]. It principally acts by interacting with the DNA base pairs, inhibiting the RNA polymerase and DNA polymerase, thus hindering DNA synthesis and altering the mitochondrial membrane and the glycoprotein structure of the endoplasmic reticulum’s surface [4,5,6].

One of the main adverse effects of this drug is tissue damage at the injection site [7] and reduced accumulation of the drug in the parasite [8]. ISM was found to be an efficient trypanocide for the therapeutic/prophylactic use against trypanosomosis; however, it has been reported to produce restiveness, hyperaesthesia, quivering, shivering, convulsions, and even death in experimental animals [9]. The other issues are related to its narrow therapeutic index and observed variations in its prophylactic activities [10]. Due to the nanosize, ISM may cross biological barriers such as cells/nucleus and other organelles, and cause adverse effects in the recipient. The nanomaterials can infiltrate the cellular components and impact the cells’ respiratory process by quieting the key enzymes due to the formation of complex structures with the catalytic sulfur of thiol-group in ‘cysteine residues’ [11,12]. Thus, it generates reactive oxygen species such as superoxide, hydrogen peroxide, and hydroxyl ions. When nanoparticles are given in high enough concentrations, they cause cytotoxic/genotoxic effects such as damage to genetic material, chromosomal abnormalities, and cell death [12]. Previously, we synthesized a novel nanoformulation, i.e., isometamidium chloride-loaded alginate/gum acacia nanoparticles (ISM SANPs), which can overcome the pitfalls associated with the ISM. The biodegradable, biocompatible polymers encapsulated the ISM, thus avoiding direct contact with cellular components and allowing the sustained release of the drug [13]. Due to nanosize, effective dose is also reduced. We have reported the remarkable cyto-compatibility of the ISM SANPs on equine blood mononuclear and erythrocytes as compared to ISM [13]. However, it is essential to address genetic safety concerns if any damage is there, as a result of ISM SANPs treatment. Apurinic/apyrimidinic (AP) sites are one of the major types of DNA lesions formed during base excision and renovation of oxidized/deaminated/alkylated-bases. It has been assessed that, by each cell about 2.00 × 10^5^, base lesions are produced daily. These lesions occur when a purine or pyrimidine base is lost from the DNA molecule, leaving a gap or an unpaired site in the DNA strand. They can interfere with DNA replication and transcription by blocking the progression of DNA or RNA polymerases. This can lead to mutations or other genomic alterations that can contribute to carcinogenesis, aging, and other diseases. AP sites can also cause cell death or senescence if they are not repaired properly. The intensity of cellular AP-sites is a valuable sign of DNA lesion/repair against chemical damage. By measuring the levels of AP sites in cells, researchers can gain insights into the mechanisms of DNA repair and the effects of various DNA-damaging agents. The present investigation determined the dose-dependent DNA damage potential of the ISM SANPs before therapeutic applications against trypanosomosis in animals. 

## 2. Materials and Methods

### 2.1. Materials

Sodium alginate (SA) and glutaraldehyde were purchased from “Sigma-Aldrich Chemicals Private Ltd. (Bangalore, India)”. Gum acacia (GA) was procured from “Qualigens Fine Chemicals Pvt. Ltd., Mumbai, India”. All other chemicals were of the analytic mark and acquired from “Sigma-Aldrich Chemicals Private Ltd., Bangalore, India”, except isometamidium chloride, which was procured from Intas Pvt. Ltd. The ‘comet-slides’ were procured from “Invitrogen, Life technologies (Carlsbad, CA, USA/Chromous Biotech, Bangalore, India)”. Reagents of analytical grade were utilized in all the assays/experiments. 

### 2.2. Fabrication of ISM-Loaded Nanoparticles

We synthesized ISM SANPs by drop-wise addition of an aqueous solution of 0.5 mg/mL gum acacia (GA) into an aqueous solution of 1% sodium alginate (SA), containing isometamidium chloride hydrochloride (Nyzom^®^, Intas, Ahmedabad, India) ISM@15 mg/mL under constant stirring up to 2 h using cross-linker glutaraldehyde, as described previously [13]. ISM-laden SA and GA nanoparticles (ISM SANPs) were collected by centrifugal force at 12,000× *g* for 45 min. and washed twice with deionized water to get rid of unloaded ISM. The precipitated NPs were again coated with mannitol (1.5% *w*/*v*) and lyophilized at −900 °C and 0.0010 mbar pressure. Dummy nanoparticles were fabricated similarly but without ISM. The concentration of isometamidium chloride (ISM) in ISM SANPs solution was calculated using the supernatant obtained after centrifugation of the ISM SANPs. It was calculated by the regression equation obtained by preparing a standard curve for various drug concentrations using a UV spectrophotometer [13].

### 2.3. Morphology/Size

The NPs obtained in Section 2.2 were mixed in distilled water to assess the morphology/size of ISM SANPs by “transmission electron micrography (TEM) (Morgagni 268D, Fei Electron Optics, Eindhoven, The Netherlands)” using high-contrast image method with a 100 kV speed. The FTIR analysis of isometamidium chloride-loaded alginate gum acacia nanoformulation (ISM SANPs) and unloaded alginate gum acacia nanoformulation were performed by using an FTIR spectrophotometer (Nicolet iS50 FTIR, Thermo Fisher Scientific, Waltham, MA, USA) for the identification of functional groups involved in synthesis of ISM SANPs at wavelengths ranging from 4000 cm^−1^ to 500 cm^−1^.

### 2.4. Cell-Culture Conditions

The Vero cell line (African green monkey kidney cell line) was obtained from National Centre of Veterinary Type Cultures, National Research Centre on Equines, Hisar, India and established in ‘Eagle’s minimum essential medium (EMEM)’ with an additional 10% (*v*/*v*) fetal bovine serum, ‘HEPES 10 mM, L-glutamine 2 mM, sodium bicarbonate 25 mM’ and antibiotic/antimycotic solution (penicillin 10,000 units/mL, streptomycin-10 mg/mL, amphotericin B-25 µg/mL). The cells were cultured at 37 °C in a 5% CO_2_ incubator. ‘HeLa cells’ grown in supplemented ‘Dulbecco’s modified Eagle’s medium (DMEM)’ (containing 10% fetal bovine serum, 50 µg/mL ‘streptomycin sulfate, 10 µg/mL gentamicin sulfate, 2 mM L-glutamine and 50 µM 2-mercaptoethanol) were incubated at the same conditions.

### 2.5. Cytoviability/Toxicity Studies

The cytotoxic/viability studies of ISM SANPs were performed on African monkey kidney cells (Vero cells) by an objective quantitative test using “resazurin” dye]. Vero cells @ 1 × 10^4^ were grown in a 100 µL volume of growth medium in a 96-well plate at 37 °C temperature in CO_2_ (5%) incubator until 70% confluence was achieved. Subsequently, these confluent cells were stimulated with serially diluted concentrations of ISM, ISM SANPs starting from 1000 µg/mL down to 31.25 µg/mL, and dummy NPs, and were cultured another time for 24 h at the conditions specified above. Resazurin @ 1 mg/mL prepared in DMEM in a volume of 10 µL was pipetted into each sample and kept for 4 h at the same conditions. Following four hours, the pink-colored “resorufin” was generated proportionally related to the metabolically active cells. The optical density was recorded spectrophotometrically using an “ELISA plate-reader, Power Wave XS2, CA, USA) at wavelength 590 nm. IC_50_ was calculated using a four-parameter logistic regression model, Y = Min + (Max − Min)/1 + (X/1C50) Hill coefficient using an online tool [14].

### 2.6. Isolation of Peripheral Blood Mononuclear Cells and Treatments

Peripheral blood mononuclear cells (PBMCs) of the Marwari horse were separated by density gradient centrifugation on histopaque (Sigma, St Louis, MO, USA) as described previously [15]. The viable cells (1 × 10^7^) were counted by an automatic cell counter (Invitrogen, Waltham, MA, USA), and re-suspended at 10^7^ cells per mL in EMEM (supplemented with 10% fetal bovine serum, 50 µg/mL ‘streptomycin sulfate, 10 µg/mL gentamicin sulfate, 2 mM L-glutamine and 50 µM 2-mercaptoethanol’. The cells were given treatments of varied concentrations of ISM and ISM SANPs (100 µg/mL, 50 µg/mL, and 25 µg/mL), and untreated cells were kept as control. Cells were incubated in six-well round bottom plates using a culture medium at 37 °C in 5% CO_2_ and 95% humidity.

### 2.7. Isolation of DNA

DNA extraction of treated cells with different concentrations of ISM and ISM SANPs (100 µg/mL, 50 µg/mL, and 25 µg/mL) and untreated cells was performed as per the M/S protocol. Briefly, the respective specimens were centrifuged for 5 min at 300× *g* and the cell pellets were re-suspended in 200 µL PBS each. This sample mixture was combined with an equal volume of lytic buffer and proteinase K in 20 µL quantity each, and incubated for 10 min at 56 °C. This was followed by the addition of absolute ethanol in 210 µL volume. After proper mixing using a vortex, the samples were subjected to QIAamp spin column and we discarded the flow-through resulting from brief spinning using microcentrifuge (Tarsons, SPINWIN™ MC03, Kolkata, India). Washing was performed with the buffers supplied with the kit using a short spin. Finally, 100 µL elution buffers were pipetted gently directly to the column matrix and kept at room temperature for 5 min, and DNA was collected by centrifugal force at 8000× *g* for one min. The quality and quantity were determined by a Nanodrop spectrophotometer (Eppendorf, D30 Biophotometer, Darmstadt, Germany).

### 2.8. Estimation of Apurinic/Apyrimidinic (AP) Sites to Examine DNA Damage

DNA damage was assessed by colorimetric assay using DNA Damage kit (Abcams, Cambridge, UK). The assay uses an aldehyde reactive probe that reacts with an aldehyde group on the open ring of AP sites which was tagged with biotin and quantified by streptavidin enzyme conjugate. The PBMCs were treated with ISM, ISM SANPs, and dummy NPs for 24 h and 48 h. DNA was extracted from treated and untreated PBMCs using a DNeasy/isolation kit (Qiagen, Hilden, Germany) as per the manufacturers’ guidelines. For estimating DNA damage, 500 ng of purified DNA of treated and untreated PBMCs at different time hours (24 h and 48 h) were mixed with 5 μL aldehyde reactive probe (ARP) for each sample at the base of a centrifuge microtube and incubated at 37 °C for 1 h to annex the DNA AP area. Following the addition of 88 μL TE and 2 μL glycogen to each reaction solution, it was thoroughly mixed and 0.3 mL of absolute ethanol was added to each sample, mixed well, and kept at −20 °C for 10 min. The AP-site tagged DNA was precipitated by centrifuging at 13,000 rpm for 10 min. Pellet was washed thrice with 0.5 mL 70% ethanol and air-dried for 5 min to remove the trace amounts of ethanol. The biotin-tagged DNA samples were prepared in 1 mL of TE buffer. The ARP-labeled DNA samples and ARP-DNA standards in 60 μL quantity were pipetted into the designated wells of 96-well plates in duplicate. Following the addition of 100 μL of the DNA binding solution, the plate was incubated at room temperature overnight to allow the tagged DNA to bind on the plate surface. The next day, after discarding the DNA binding solution and washing five times, 100 μL of HRP-Streptavidin solution was added, mixed at room temperature for one hour, and washed as described above, followed by the addition of 100 μL of horse radish peroxidase enzyme as a developer; then, it was incubated for 1 h at 37 °C. The reaction was stopped by 1 M sulphuric acid and absorbance was measured at 450 nm to quantitate the AP sites.

### 2.9. Statistical Evaluation

The results are shown as the mean and standard deviation (SD) of at least three separate experiments. Student’s test was used to compare the differences between experimental groups using the online Graphpad calculator (http://www.graphpad.com/quickcalcs/ttest1.cfm, accessed on 17 November 2022). At *p* 0.05, significant differences were taken into account.

## 3. Results and Discussion

### 3.1. Synthesis and Morphology of ISM-Loaded Sodium Alginate and Gum Acacia Nanoparticles

**Figure 1:**
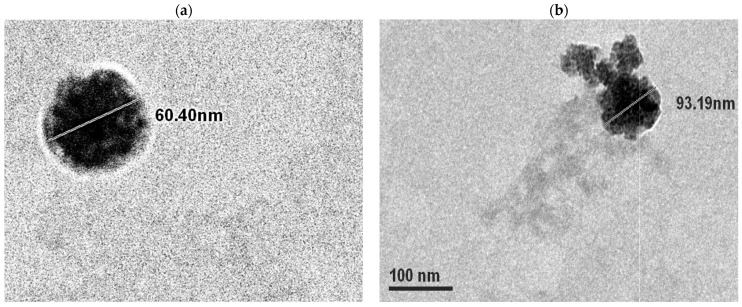
TEM image of ISM SANPs displaying size and morphology of NPs (60.4 nm and 93.19 nm diameters in figures (**a**,**b**), respectively. The polymeric covering of white color is also depicted in these figures. (**b**) shows ISM release).

A successful nanoformulation must generally meet the criteria of nanoparticle size, high drug encapsulation efficiency, and a well-dispersed state. In our study, ISM SANPs were well-formed and regular in shape. TEM micrograph of the ISM SANPs depicted a shell formation with layers where the internal core is coated with SA/GA polymer casing. The dimensions of ISM SANPs in the representative Figure 1a,b are shown as about 60.4 nm and 93.19 nm diameters, respectively, suggesting that the particle size of the formulation is less than 100 nm. The polymeric covering of white color is also depicted in these figures. ISM SANPs contained 5.93% drug, i.e., 10 mg of synthesized nanoparticles contains 0.593 mg of ISM, respectively.

The FTIR spectra of the alginate gum acacia nanoformulation with and without loading of ISM show differences in peak intensities and positions. The peak at 3227.72 cm^−1^ in the loaded formulation is slightly shifted from the peak at 3189.80 cm^−1^ in the unloaded formulation, which both possibly correspond to the OH group. This shift may suggest the formation of hydrogen bonds between the ISM and the nanoformulation components. The peaks at 2935.16 cm^−1^ and 2935.85 cm^−1^ in both formulations correspond to C-H stretching, indicating the presence of aliphatic chains in the samples. The peak at 1607.52 cm^−1^ in the unloaded formulation indicates the presence of C=C stretching in a conjugated alkene, while the peak at 1596.30 cm^−1^ in the loaded formulation suggests the presence of aromatic C=C stretching. This change in peak position may also indicate the interaction between the ISM and the nanoformulation, as the ISM contains aromatic rings that could be interacting with the formulation.

**Figure 2:**
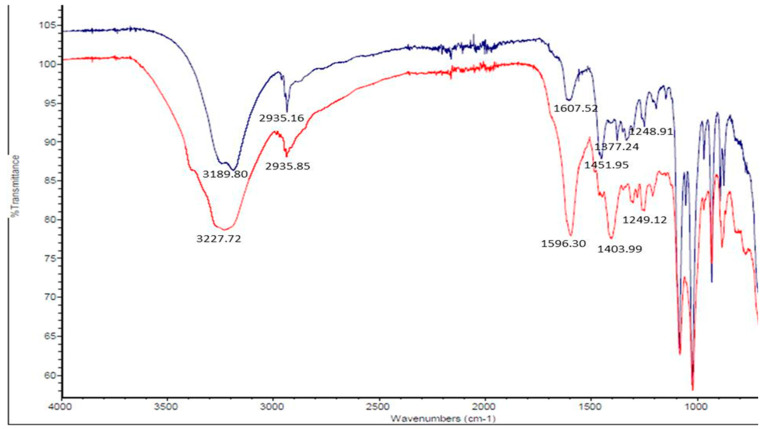
The FTIR analysis of isometamidium chloride-loaded alginate gum acacia nanoformulation (ISM SANPs: red) and unloaded alginate gum acacia nanoformulation (blue).

The peaks at 1451.95 cm^−1^ in the loaded formulation may indicate the presence of C=O stretching in the ISM, while the peaks at 1377.24 cm^−1^, 1248.91 cm^−1^, and 1249.12 cm^−1^ in both formulations suggest the presence of C=O stretching in the SA and GA components. Overall, the differences in peak positions and intensities between the loaded and unloaded formulations suggest that there is an interaction between the ISM and the nanoformulation, which may be important for the development of effective drug delivery systems (Figure 2).

### 3.2. Cytotoxicity of ISM SANPs on Vero Cells

**Figure 3:**
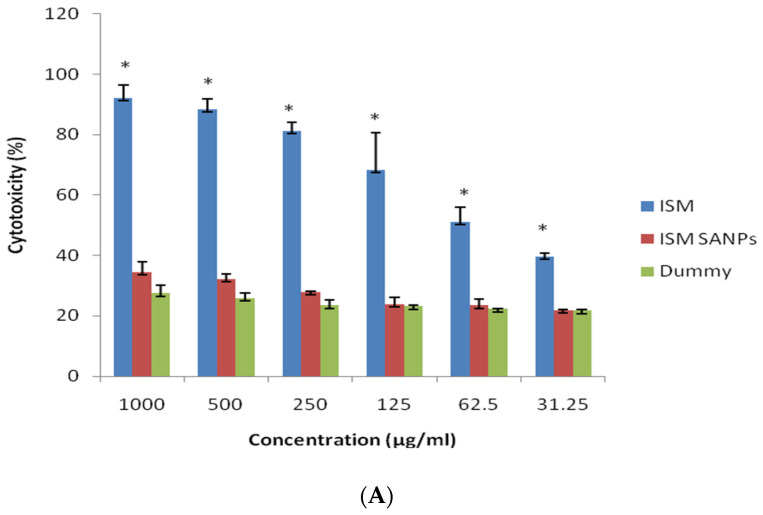

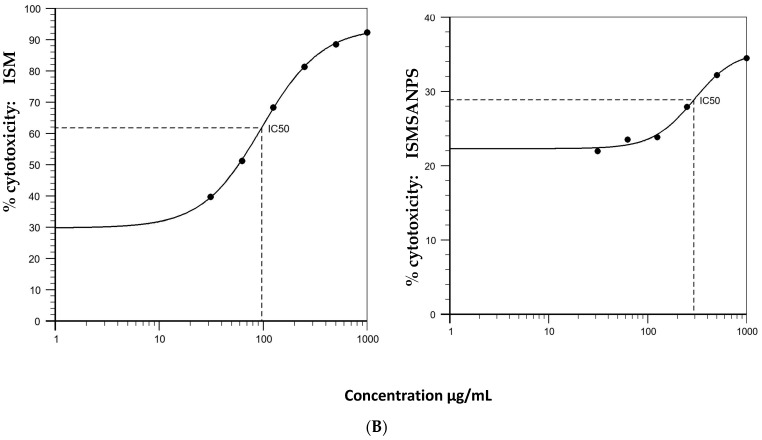
(**A**) Cytotoxicity in response to different concentrations of ISM, ISM SANPs, and dummy NPs is expressed relative to untreated Vero cells, as determined by resazurin assay. Error bars show the standard deviation. The variation in toxic effects amongst ISM and ISM SANPs/controls was statistically significant (*p* < 0.01) shown by *. (**B**) IC_50_ determined for ISM and ISM SANPs. IC_50_ was calculated using a Hill four-parameter logistic regression model.

Nanoparticles can have an unfavorable impact on cellular structures owing to their astonishing physicochemical properties. Cytocompatibility assays are key inspections for appraisal of biosafety and description of the cells’ reaction to the therapeutic candidate and provide information on how the NPs alter their growth and metabolism. Here, we report the concentration-based cytocompatibility or toxicity of ISM SANPs corresponding to their metabolic function. The cytotoxic activities of ISM SANPs and ISM were assessed by quantitative assay via ‘resazurin’ dye. Isometamidium chloride exhibited higher cytotoxicity than ISM SANPs at varied doses (Figure 3A). However, cell culture with dummy NPs containing GA/SA without ISM, and ISM SANPS showed good cytocompatibility without any significant difference (*p* > 0.01, 0.1528–0.5337). The variation in toxic effects amongst ISM and ISM SANPs/controls was statistically significant (*p* < 0.01 (0.0045–0.0021). These data were plotted and Hill 4 parameter sigmoidal regression was performed. IC_50_ calculated for ISM and ISM SANPs were 96.686 µg/L and 292.260 µg/mL, respectively (Figure 3B).

### 3.3. Apurinic/Apyrimidinic (AP) Sites Produced by ISM and ISM SANPs

**Figure 4:**
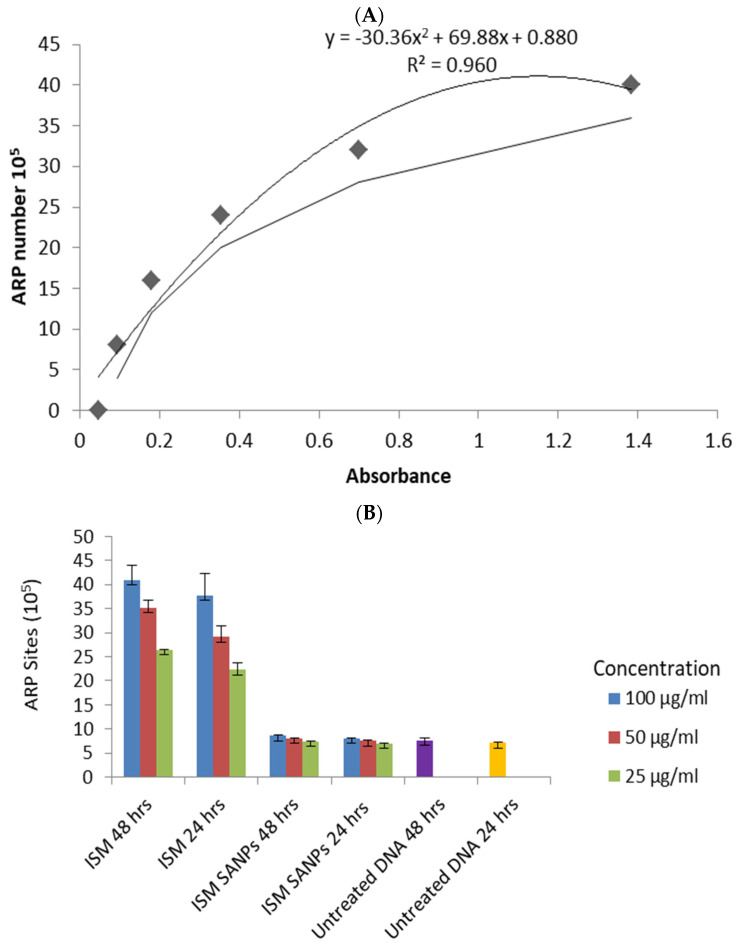
(**A**) Standard curve prepared using DNA standards containing different ARP concentrations on the *x*-axis against the absorbance at wavelength 450 nm on *y*-axis. (**B**) ARP sites of PBMCs in response to ISM and ISM SANPs treatments at 24 h and 48 h along with untreated cells. ARP sites of untreated DNA at 48 h and 24 h are shown by violet and yellow bars, respectively. Error bars show the standard deviation. DNA damage activity between ISM and ISM SANPs treatments was found to be statistically significant.

Apurinic/apyrimidinic (AP) sites are a type of DNA lesion that are formed during the process of base excision repair (BER), which is a mechanism that cells use to remove damaged or incorrect bases from DNA and replace them with new, undamaged bases [16]. BER is activated in response to various types of DNA lesions, including those caused by chemical or physical damage, such as oxidation, deamination, or alkylation [17]. When a base is removed or modified in some way, it leaves a gap or “hole” in the DNA backbone, which is called an AP site [18]. AP sites are considered to be an important indicator of DNA damage and repair, as they are formed in response to various types of DNA lesions [19]. They are also thought to be involved in the development of cancer and other diseases, as they can lead to mutations and other genetic abnormalities if they are not repaired properly [20]. Therefore, measuring the amount of AP sites in a sample of DNA can provide valuable information about the level of DNA damage and the effectiveness of DNA repair processes in a given system [21]. The researchers reported cytotoxicity/safety studies of the polymeric as well as lipid nanoformulations of isometamidium-loaded nanoformulation nanoparticles on peripheral blood mononuclear cells [13,22], but the genotoxicity of these formulations compared to the conventional drug has not been addressed yet. In the present study, we thought it pertinent to investigate the dose-dependent genotoxic potential of the ISM SANPs. ‘Apurinic/apyrimidinic (AP) site’ is a key indicator of deterioration of genetic material (DNA) created during base excision and repair of oxidized/deaminated/alkylated bases [23]. A standard curve was prepared using DNA standards containing different ARP concentrations on the *x*-axis against the absorbance at wavelength 450 nm on the *y*-axis to calculate ARP sites in treated and untreated DNA samples showing 0.96 R^2^ (regression coefficient) using polynomial fit (Figure 4A). It was demonstrated that ISM produced average ARP sites 40.93, 35.11 and 26.33 (×10) at 100, 50, 25 µg/mL concentrations, respectively, after 48 hrs of exposure, whereas ISM SANPS yielded 8.61, 8.12, and 7.45 (×10) ARP sites at these concentration for the same exposure (Figure 4B). Similarly, we observed mean 37.80, 29.09, 22.24 (×10) ARP sites on ISM exposure for 24 hrs at these concentrations, respectively, whereas ARP sites were drastically reduced (8.06, 7.53, and 6.98 (×10) ARP sites) at the corresponding concentration and time. DNA damage as shown by ARP sites between ISM and ISM SANPs treatments was found to be statistically significant. At all the concentrations (100, 50, and, 25 µg/mL) at 48 h and 24 h of treatments, we observed a statistically significant difference in DNA damage activity between ISM and ISM SANPs at (*p* < 0.05) at 95% of confidence, as shown in Table 1. However, we could not find any statistically significant difference between ISM SANPs and untreated cells.

In conclusion, ISM is a trypanocide, a type of drug used to treat and prevent animal trypanosomosis, a disease caused by parasites that affects livestock worldwide. It works by inhibiting DNA synthesis and altering the structure of certain cellular components, but it can also produce adverse effects such as restlessness, convulsions, and death in experimental animals. It has a narrow therapeutic index and its prophylactic activities can vary. We have developed a nanoformulation of ISM called ISM SANPs, which is made of biodegradable, biocompatible polymers that encapsulate the drug and allow for sustained release. However, it is important to assess the genetic safety of ISM SANPs before it can be used therapeutically. The present study investigates the dose-dependent DNA damage potential of ISM SANPs in order to better understand its potential risks and benefits. This study showed concentration-reliant cytotoxicity or genotoxicity in cells after ISM, ISM SANPs treatments. Isometamidium chloride exhibited higher cytotoxicity than ISM SANPs at varied doses. However, cell culture with dummy NPs containing GA/SA without ISM and ISM SANPs showed good cytocompatibility without any significant difference. Apurinic/apyrimidinic, a key indicator of deterioration of DNA produced during base excision and repair of oxidized/deaminated/alkylated bases against chemical damage, revealed a statistically significant difference in DNA damage activity between ISM and ISM SANPs. However, we could not find any statistically significant difference between ISM SANPs and untreated cells. ISM SANPs have been demonstrated to be innocuous at all tested concentrations, including the highest concentration, which implies that they are safe to use. This finding has significant implications for potential future use of the ISM SANPs in drug delivery systems, as it means that effective concentrations of the ISM can be reduced while still achieving the desired therapeutic effect. Moreover, the continued availability of ISM is an added advantage, and by using the ISM SANPs, the availability and efficacy of the ISM can be sustained for longer periods, leading to potentially more efficient and effective drug delivery. Overall, this study’s findings can pave the way for the development of new and improved drug delivery systems of ISM that are safer, more cost-effective, and more efficient. However, further studies are justified to investigate its potential against economically important multispecies trypanosomosis.

## Figures and Tables

**Table 1:** The difference between ARP sites of ISM amd ISM SANPS treatments.

Time (Hours)	Concentration (µg/mL)	Statistically Significant Difference	Two-Tailed *p* Value for ARP Sites for ISM amd ISM SANPS
48 h	100	✓	0.0044
	50	✓	0.0020
	25	✓	0.0001
24 h	100	✓	0.0113
	50	✓	0.0205
	25	✓	0.0053

## Data Availability

Not applicable.
